# Human amniotic mesenchymal stem cells to promote/suppress cancer: two sides of the same coin

**DOI:** 10.1186/s13287-021-02196-x

**Published:** 2021-02-12

**Authors:** Ameneh Jafari, Mostafa Rezaei-Tavirani, Behrouz Farhadihosseinabadi, Hakimeh Zali, Hassan Niknejad

**Affiliations:** 1 Department of Basic Sciences, Faculty of Paramedical Sciences, Shahid Beheshti University of Medical Sciences, Tehran, Iran; 2 Proteomics Research Center, Faculty of Paramedical Sciences, Shahid Beheshti University of Medical Sciences, Tehran, Iran; 3 Hematopoietic Stem Cell Research Center, Shahid Beheshti University of Medical Sciences, Tehran, Iran; 4 Department of Tissue Engineering and Applied Cell Sciences, School of Advanced Technologies in Medicine, Shahid Beheshti University of Medical Sciences, Tehran, Iran; 5 Department of Pharmacology, School of Medicine, Shahid Beheshti University of Medical Sciences, Tehran, Iran

**Keywords:** Amniotic mesenchymal stem cell, Tumor suppressing, Tumor promoting, Angiogenesis, Apoptosis

## Abstract

Cancer is a leading cause of death in both developed and developing countries, and because of population growth and aging, it is a growing medical burden worldwide. With robust development in medicine, the use of stem cells has opened new treatment modalities in cancer therapy. In adult stem cells, mesenchymal stem cells (MSCs) are showing rising promise in cancer treatment due to their unique properties. Among different sources of MSCs, human amniotic fluid/membrane is an attractive and suitable reservoir. There are conflicting opinions about the role of human amniotic membrane/fluid mesenchymal stem cells (hAMSCS/hAFMSCs) in cancer, as some studies demonstrating the anticancer effects of these cells and others suggesting their progressive effects on cancer. This review focuses on recent findings about the role of hAMSCs/hAFMSCs in cancer treatment and summarizes the suppressing as well as promoting effects of these cells on cancer progression and underling mechanisms.

## Introduction

Cancer is one of the leading causes of mortality worldwide [1]. Over the past decades, numerous studies have been conducted to find new therapeutic approaches with greater effectiveness and fewer side effects to replace conventional therapies [2]. Despite advancing progresses in the cancer survival rate, many conventional treatments need to be replaced by new and innovative ones. In this light, great potential of stem cells underscores their therapeutic index in treatment of cancers. In particular, numerous experimental studies have highlighted the mesenchymal stem cells (MSCs) as promising tool in cancer therapies [3–5].

The discovery of mesenchymal stem cells (MSC), as the non-hematopoietic stem cells, goes back to1960s when Friedenstein and colleagues isolated these cells from the bone marrow [6]. MSCs are naturally developed in the human body and can be easily obtained from a number of adult and fetal tissues such as the heart, liver, kidney, skeletal muscle, adipose and connective tissue, synovial tissue, placenta, and umbilical cord [7]. These cells express some specific cell markers like CD73, CD90, and, CD105; however, they do not express CD34, CD45, CD14, CD11b, CD79-a, CD19, and HLA-DR [8]. MSCs are known as multipotent cells with a great potential of self-renewal and differentiation into a versatile cell linage such as adipocytes, chondrocytes, myocytes, osteocytes, fibroblasts, neurons, and epithelial cells [9, 10]. In general, MSCs show intrinsic antitumor property due to their anti-proliferative activity, induction of apoptosis, and suppression of angiogenesis [11]. However, it is not fully understood how these cells govern their anti-cancer effects. Moreover, the anti-cancer effect of MSCs seems to be cancer type-related. For example, bone marrow-derived MSCs stimulated osteosarcoma, breast cancer, colorectal tumor, and gastric cancer, while those isolated from the adipose tissue or umbilical cord showed inhibitory effect on a group of cancer types including lung, liver, glioma, and prostate cancers. The rationale behind this discrepancy remains unclear; however, some reasons have been proposed such as lack of a standard experimental method, challenges with isolation of MSC subgroups due to the lack of specific markers, and unpredictable behaviors of these cells resulting from the strong influences of microenvironment surrounding them [12].

To date, most preclinical studies have focused on bone marrow-derived MSCs, although these cells might not be the best available source. The bone marrow-derived MSCs have been used in various clinical therapies to treat several cancer types including glioma, ovarian, and prostate cancers [13]. However, using these cells faces with some challenges including (i) need for aggressive operations to harvest them from the bone marrow (ii) limited number of the isolated cells (iii) and decreased ability to differentiate as the patient ages [14]. There are other accessory sources for harvesting MSCs such as the adipose tissue, umbilical cord, and Wharton’s jelly [15, 16]. Adipose derived-MSCs are highly suitable alternative due to ease of tissue collection with higher number and favorable proliferation capacity in vitro compared with the other sources [17]. MSCs derived from the umbilical cord blood and Wharton’s jelly are also isolated through a completely non-invasive and simple procedure [18, 19]. In recent years, the MSCs derived from the amniotic fluid and amniotic membrane have been introduced as an attractive and potent stem cell sources for clinical application because of their easy, safe, and painless collection procedures with minimized ethical issues. In recent years, placenta-derived MSCs, especially those isolated from human amniotic membrane (hAMSCs) and amniotic fluid (hAFMSCs), are more popular due to their non-invasive isolation techniques, large-scale supply, genome stability, non-tumorigenic, being able to interact with different tissue environments, and acceptable ethical issue [20, 21].

Until now, various studies have been conducted to evaluate the effect of hAMSCs/hAFMSCs on cancer cell behaviors. Some studies have revealed that hAMSCs/hAFMSCs induced tumor growth and metastasis [22]. However, reports confirming the anti-cancer effects of these cells are also available in literature [23, 24]. The controversial results and poor understanding of mechanisms behind the effect of hAMSCs/hAFMSCs on cancer cells hamper those researchers who are trying to use such cells in clinic [25]. This review article highlights recent findings regarding the effect of hAMSCs/hAFMSCs on cancer cells and complies evidences supporting both anticancer and cancer growth promotor effect of these cells.

## MSCs derived from the amniotic fluid and amniotic membrane

**Fig. 1:**
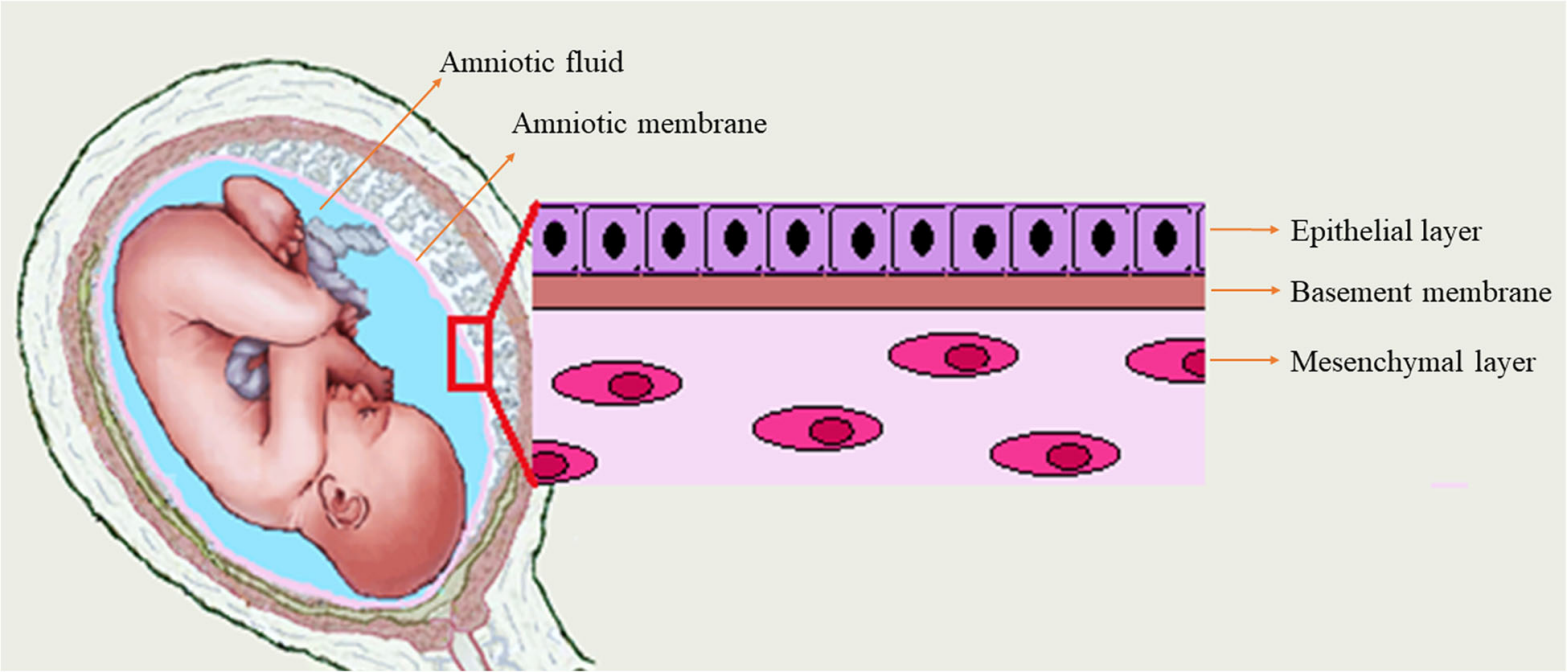
Schematic illustration of amniotic fluid, amniotic membrane, and its three major layers

Amniotic fluid (AF) fills the amnion cavity that surrounds the fetus and serves as the protective and provider fluid, ensuring the fetus to earn required protection and nutrients during embryogenesis [26, 27] (Fig. 1). The AF is mainly composed of water, chemical substances, and cells. In the early twentieth centuries, it is found that AF contains abundant stem cells [27]. Across various cellular subpopulations of AF, amniotic fluid mesenchymal stem cells (AFMSCs) are particularly favorable because of their therapeutic potential. These cells can be easily collected by amniocentesis in the second and third trimesters or even at the end of pregnancy [28, 29]. It not only provides a safe way to isolate these cells with a minimum risk for the mother and fetus, but also avoid any ethical issues that normally found in work with embryonic stem cells [24, 30]. These cells express the stem cell markers including Oct-4, c-Myc, Sox2, Nanog, and SSEA3 that well-confirm their pluripotency [27, 31, 32]. In addition to the pluripotency markers, AFMSCs exhibit high levels of several MSC markers including CD29, CD44, CD73, CD90, CD105, CXCR4, stromal cell-derived factor 1 receptor (SDF-1), CD146, CD166, and CD184 [32]. Regarding the pluripotent capacity of AFMSCs, they are able to differentiate into adipogenic, chondrogenic, and osteogenic lineages under a specific condition [33, 34].

Amnion or human amniotic membrane (hAM) is the innermost layer of fetal membranes, which is directly in contact with the amniotic fluid and the fetus. The hAM consists of three major layers include the epithelial layer, the basement membrane, and the mesenchymal layer [29, 35] (Fig. 1). Human amniotic mesenchymal stromal cells (hAMSCs) can be harvested from the mesenchymal layer of hAM by a simple enzymatic digestion procedure ended to isolate about 10^7^ cells from a single hAM [36, 37]. Similarly to MSC, hAMSCs are pluripotent stem cells and able to differentiate into the three germ layers [38]. hAMSCs express specific surface markers such as CD14, CD29, CD44, CD49, CD90 (Thy-1), CD105 (endoglin), CD166, and SSEA-3/4 [39], while they do not express HLA-DR (MHC class II), CD19, CD45, and CD133 [32]. RT-PCR results also confirmed that these cells expressed stem cell markers like SSEA-4, Nanog, and OCT4 [40].

Stem cell properties of AMSCs/AFMSCs along with their low immunogenicity introduce them as a promising source for applications in the field of tissue engineering, regenerative medicine, and cell therapy [41, 42]. Some unique features of AMSCs/AFMSCs that make them the suitable candidates for therapeutic care diseases ranging from wound healing to cancer therapy are listed in Table 1 [43, 44].

**Table 1:** The unique characteristics of amniotic fluid/membrane mesenchymal stem cells [43, 44]

Feature	Description
Accessibility	No need to use invasive procedures for collection
Availability	Readily available in large number for transplantation
Plasticity	Ability to differentiate into different cell types
Mitotic stability	Karyotype stability during several cell divisions
Ethical acceptability	Use without ethical concerns.
Low-risk	No need to be concern regarding transplant-related diseases, such as immunoreaction and cancer

## Tumor-suppressing effects of hAMSCs/hAFMSCs

### Proapoptotic effects of hAMSCs/hAFMSCs

MSCs are strong proliferation inhibitors of immune cells like the natural killer cells (NK cells). The same results have been reported on numerous cancer types such as breast cancer [45], hepatoma [46], ovarian cancer [23], and glioma [47]. It is reported that MSCs exert their growth inhibitory effect through MSC-secreted prostaglandin E2 (PGE2), IL-6, indoleamine2,3- dioxygenase (IDO), TGF-β1, and NO [48, 49]. Recently, Aziz et al. found a significant reduction of survival rate in the ovarian cancer cells after co-culturing with hAFSCs compared with the control group [23]. The authors reported a significant increase in the expression of P53 and P21 genes, and also, the genes involved in apoptosis in the treated cancer cells. In that study, the expression rates of cyclin D1 and B1 genes were lower than the control group, indicating blockage of cell cycle progression from phase G0 to G1 and inhibition of phase S commencement [23]. hAMSCs are not only able to inhibit the proliferation of immune cells [50] but also they promote the apoptosis of B and T lymphocytes [51]. Kang et al. showed a significantly decrease in the growth and proliferation of cancer cells in vitro and in vivo. Through evaluating the cytokines involved in this phenomenon, they pointed out the key roles of hAMSC-released factors such as TNF-α, TGF-β, TNF-β, and IFN-γ as the proapoptotic agents [52].

Aquaporins, which serve as channels involved in the transfer and control the water contents in cells [53], are also responsible for the anti-proliferative and proapoptotic activities of the AM cells [54]. Indeed, activation of peroxisome proliferator-activated receptor-γ (PPAR-γ) signaling by AM ingredients resulted in the upregulation of aquaporins and consequently apoptosis in cancer cells [55, 56].

In a study, intravenous administration of amniotic mesenchymal cells in glioma C6 tumors in a single and multiple doses reduced the tumor size by approximately 30 and 50%, respectively. High expression of caspase-3, caspase-8, and Bax, and low expression of Bcl-2 in tumor cells after treatment with amniotic mesenchymal cells confirmed the apoptosis as the main mechanism of cancer cell death. You et al. reported that injection of genetically modified hAFMSCs expressing IL-2 led to induction of apoptosis in the ovarian cancer nude mice models [57].

### Anti-angiogenesis effects of hAMSCs/hAFMSCs

Given the necessity and importance of angiogenesis for tumor growth and metastasis, controlling of angiogenesis in tumors is an exciting strategy to inhibit cancer progression [58]. In contrast to some reports claiming the release of angiogenic factors by MSCs, Lee et al. observed that MSC-derived extracellular vehicles (EVs) inhibited angiogenesis in tumor cells through downregulation of VEGF [59]. MSC-derived exosomes contain a wide range of proteins and RNA, reflexing the nature of the cells producing them. Among these mediators, miR-16 which is known for targeting VEGF, have been enriched in MSC-derived exosomes that partly explained the antiangiogenic effect of these vesicles [60].

In a paracrine manner, hAMSCs secrete mediators like thrombospondin, TIMP 1–4, pigment epithelium-derived factor (PEDF), collagen XVIII, and endostatin that contribute in suppressing cancer cell migration and cancer angiogenesis [61, 62]. Downregulation of factors such as VEGF, PDGF-AA, PDGF-BB, BMP-4, and CXCL16 has been reported after co-culturing of cancer cells with MSCs. These results have been related to the anti-angiogenesis and anti-migration effects of MSCs [63–65]. Meng et al. observed similar results following co-culture of SPC-A-1 cells with hAMSCs [66].

HIF1α, a client protein of HSP90, involves in activation of VEGF [67, 68]. In addition, HIF1α by activating MMP-2 induces invasion and metastasis of tumor cells [69]. The crucial role of HSP90 in stabilization and activation of HIF1α has been proved. It seems that inhibition of HSP90 is one of the anticancer mechanisms of amniotic stem cells [70].

### Anti-inflammatory effects of hAMSCs/hAFMSCs

Inflammation is a process involving the activation, recruitment, and function of innate and adaptive immune cells. This process plays critical roles in host protection against pathogens, tissue repair, regeneration, and remodeling, and it is essential for regulating tissue homeostasis [71]. Chronic inflammation prompts cancer development through different mechanisms. Indeed, tumors have usurped the pathways built to mediate immunity to infection and promote tissue homeostasis to their advantage [72]. In order to form an inflammatory tumor microenvironment, cancer cells, surrounding stromal and inflammatory cells, participate in a well-orchestrated reciprocal interaction [73]. It has been shown that hAMSCs have immunosuppressive activities because of their ability to secrete HLA-G, their low expression of MHC class I, and no expression of MHC class II [74–76]. These cells are able to reduce inflammatory response inhibit inflammatory cell infiltrate; reduce migration, recruitment, and activity of a broad range of immune cells; and suppress neutrophil extracellular traps [76, 77]. In a study, hAMSCs were used in rabbit corneal alkali-burn models. The obtained result indicated that intracameral hAMSC injection could induce an anti-inflammatory and anti-fibrotic environment and subsequently stimulate corneal wound healing [76]. Based on evidence, the hAMSC and hAMSC-derived conditioned medium (hAMSC-CM) possess the ability to suppress T cell function, decrease the expression of Th1 and Th17 markers, increase T-regulatory (Treg) cells, and decrease NK cell cytotoxicity [78, 79]. Moreover, different studies have provided evidence of the antiproliferative effects of hAMSC-CM on lymphocytes and monocytes [77].

Studies have shown that the levels of proinflammatory cytokines are decreased by hAMSCs such as, tumor necrosis factor α (TNF-α), interleukin (IL)-1β, IL-6, IL-12, migration inhibitory factor (MIF), monocyte chemoattractant protein-1 (MCP-1), the soluble Fas-ligand (FAS-L), TNF-related apoptosis-inducing ligand (TRAIL), and IFN-γ [77, 80]. In addition, it has been reported that hAMSCs are able to secrete or increase the production of immunosuppressive molecules such as hepatocyte growth factor (HGF), IL-10, TGF-β, and indoleamine 2, 3 dioxygenase (IDO) [77, 81].

Considering the innate ability of MSCs to migrate toward the inflamed sites, recently, hAFMSCs have been used to deliver immunoregulatory cytokines [82, 83]. For example, Zhou and colleagues applied hAFMSCs as a delivering system to transfer IFN*α* to the cervical cancer tumor site. In that study, hAFMSCs were genetically modified to overexpress IFN*α* and then intravenously injected in order to migrate toward the tumor site in the mice model. Results showed the anticancer effects of IFNα-AF-MSCs on the cervical tumors due to inhibition of angiogenesis, suppression of tumor cell proliferation, and induction of apoptosis in the tumor cells [84].

In general, it has been widely demonstrated that hAMSCs and their CM are capable of dampening in vitro inflammatory conditions by suppressing proliferation, inflammatory cytokine production, stimulatory, and cytotoxic activity of various subpopulations of immune cells, and inducing anti-inflammatory and regulatory functions of T cells and monocytes.

### Role of hAMSCs/hAFMSCs in cell cycle arrest

The cell-cycle arrest is another anti-tumor mechanism of amniotic mesenchymal cells. Previous studies have indicated that MSCs isolated from different sources such as breast tissue, adipose tissue, and human palatine tonsils exert their antiproliferative effect by inducing cell-cycle arrest in the G0/G1 phase [85, 86]. Based on microarray data, the antiproliferative effect of hAMSC is attributed to downregulation of cyclin D1, cyclin E1, cyclin H, cyclin-dependent kinase (CDK) inhibitor p15^INK4b^, and CDK inhibitor p21^Waf1/Cip1^ as well as upregulation of retinoblastoma (RB). These events finally lead to G0/G1 cell cycle arrest in the cancer cells [87]. Riedel and colleagues suggested that human amniotic membrane-conditioned medium (hAM-CM) was able to inhibit DNA synthesis, cell viability, and cell cycle progression through decreasing Cyclin D1 and Ki-67 expression and increasing p21 and p53 expression [88]. The authors also observed upregulation of anti-oncomiRs such as miR-15a and miR-210 and downregulation of oncomiRs including miR-206 and miR-145 following treatment cancer cell lines (HepG2 and HuH-7 cells) with hAM-CM [88]. Several studies reported that hAMSCs could inhibit the positive regulators of the cell cycle such as proliferating cell nuclear antigen (PCNA) and the mini-chromosome maintenance complex (MCM2, MCM4, MCM5) [87, 89]. In addition, Cullin 1 (CUL 1), which mediates the degradation of various proteins including p21, is downregulated by amniotic mesenchymal stem cells [87].

## Tumor-promoting effects of hAMSCs/hAFMSCs

**Fig. 2:**
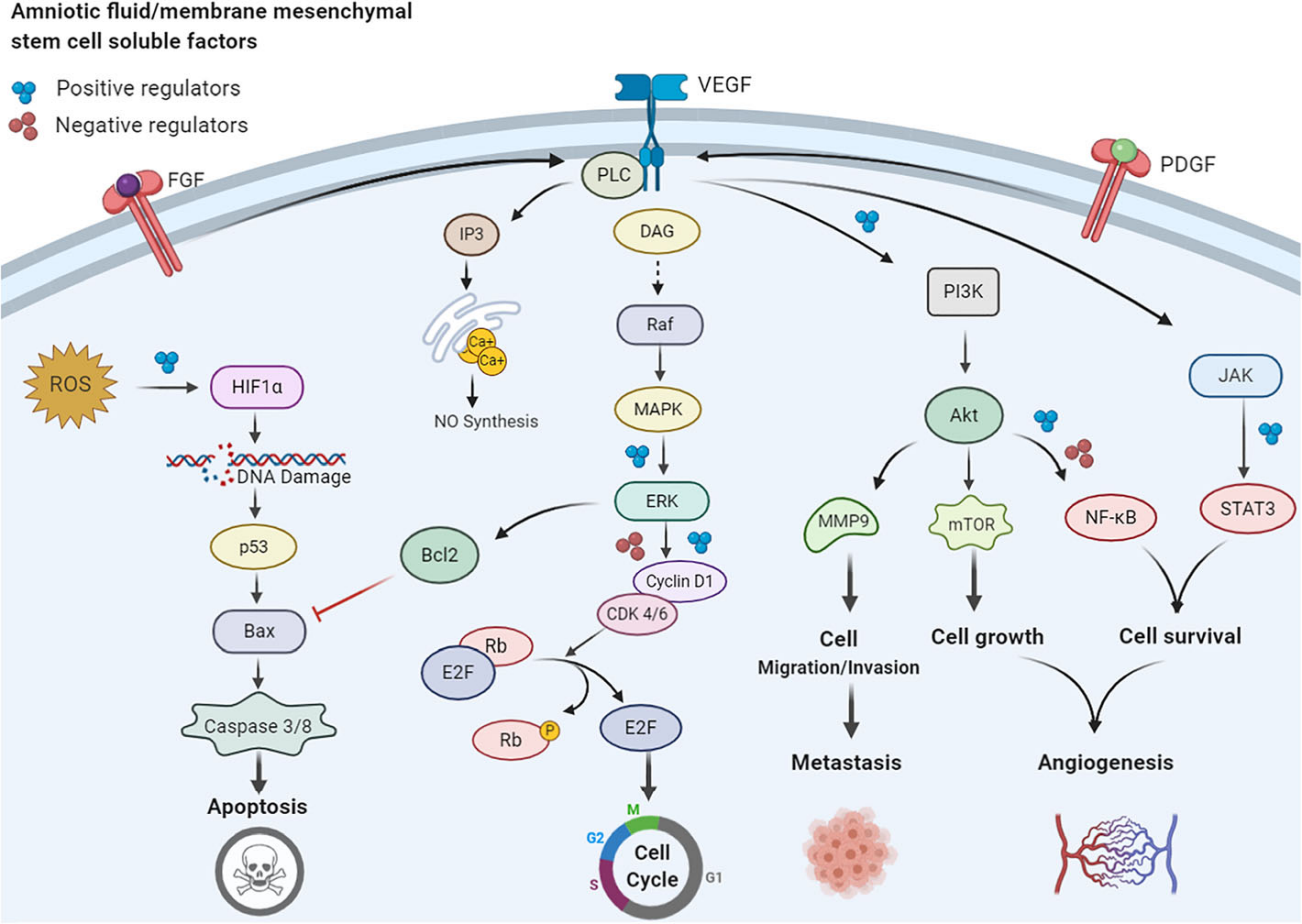
The effect of regulators secreted by hAMSCs/hAFMSCs on various cancer signaling pathways. hAMSCs/hAFMSCs produce a wide verity of mediators that affect different signaling pathways in cancer cells. As two distinct cell fate, cell proliferation and apoptosis pathways are the most important sites of action of these mediators. Proteins such as VEGF and bFGF promote angiogenesis in cancer cells, helping them to supply nutrients and scape from the tumor microenvironment. In the opposite side, the mediators secreted from these cells through activation of different signaling pathways such as PI3K/AKT signaling lead the cancer cell to apoptosis

In contrast to the anticancer effect of hAMSCs/AFMSCs that discussed above, various studies have shown that these cells promote tumor progression and metastasis by enhancing angiogenesis, upregulating Akt/mTOR signaling pathways, and promotion metastasis (Fig. 2) [90].

### Promoting angiogenesis effects of hAMSCs/hAFMSCs

Tumor angiogenesis and neovascularization are important multistep processes that occur during tumor progression and metastasis [91]. MSCs due to their potential to secrete a wide variety of growth factors, chemokines, and cytokines including VEGF, bFGF, TGF-β, MCP-1, SDF1, angiopoietin, MMPs, CXCL2, CXCL8 monocyte chemoattractant protein, IL-6, IL-8, and placental growth factor can effectively induce angiogenesis [92–94]. In this context, hAMSCs are able to promote formation, stabilization, and maturation of new vessels [91, 95]. These cells could regulate vascular network remodeling through the release of angiogenic factors or differentiation into endothelial cells (Fig. 2) [61, 96]. Studies elucidated that hAMSCs contain some angiogenic factors including VEGF, bFGF, IL-6, IL-8, MIF, growth-related oncogene (GRO), monocyte chemoattractant protein-1 (MCP-1), and intravascular adhesion molecule (ICAM). One of the most potent proangiogenic factors is VEGF that is secreted by macrophages and keratinocytes and has a crucial role in promoting proliferation of vascular endothelial cells [97]. bFGF is another strong angiogenic factor that stimulates the proliferation and migration of vascular endothelial cells [98]. Furthermore, it is shown that hAMSCs increase the expression of MMP-1 and reduce the ratio of TIMP-1/MMP-1 [99], which participates in tumor neovascularization, and subsequent metastasis [100]. In addition, hAMSCs can trigger various cell-signaling pathways needed for cell viability and neovascularization [96]. For example, hAMSCs promote angiogenesis by inducing the MAPK1/2 signaling pathway. Subsequently, the upregulation of phosphorylated ERK1/2 and RUNX2 are involved in the underlying mechanism [101]. Interestingly, hAMSC-CM could also induce neovascularization. Wu et al. found that hAMSC-CM promoted cell proliferation and angiogenesis in human aortic endothelial cells via secreted significant amounts of angiogenetic-related growth factors including VEGF, EGF, HGF, IGF-1, Ang-1, and bFGF [102]. Moreover, newly formed vascular network has been observed following treatment of human umbilical vein endothelial cells (HUVECs) with hAMSCs-CM [103]. Jeong et al. developed a gene modified hAMSC cell population overexpressing GCP-2 and SDF-1α to evaluate its angiogenic potency. The Matrigel assay revealed the high angiogenic potential of the CM obtained from the genetically engineered cells to form new vessels from HUVECs [104].

AFSCs secrete EVs containing surface mesenchymal/exosomal markers as well as angiogenic receptors such as VEGFRs. Salomon et al. revealed that EVs derived from placental MSCs promote microvascular endothelial cell migration and vascularization under hypoxic circumstances [105]. Recently, it is indicated that AFSC-derived EVs carry specific mRNA and miRNAs which are involved in different biological functions [106, 107]. Sedrakyan and colleagues found that these EVs contain modulator miRNAs including those regulate VEGF levels (miR-16.1, miR-93), VEGF receptors (miR-16.1), and positive/negative regulators of the VEGF signal transduction cascade (miR-23a, miR-27a, miR-145, miR-221, and miR-322) [108]. Moreover, the presence of a wide variety of markers on the surface or within the AFSC-derived EVs such as NOS, TGF-β, MAPK, PPAR, and Ang II probably regulate other important mechanisms involved in angiogenesis [109].

### Anti-apoptotic activity of hAMSCs/hAFMSCs

Emerging evidence suggests that tumor progression occurs in adaptation with anaerobic conditions. Indeed, chronic inflammatory status, acidic pH conditions, nutrient deprivation, and hypoxia have been observed during tumor growth [110, 111]. Under stress conditions, MSCs can maintain their characteristics and functions such as differentiation potentials and self-renewal capacity by activating autophagy pathways. Zhang et al. observed that MSC-derived exosomes could able to promote proliferation and inhibit apoptosis of skin cells after heat stress in vitro [112]. As mentioned before, MSCs are capable to release a number of pro-survival and anti-apoptotic factors such as VEGF, IGF-1/2, bFGF, PDGF, SDF-1-α, HGF, stanniocalcin-1 (STC-1), and NO [135–140]. Among them, VEGF and bFGF apply their anti-apoptotic effects by enhancing BCL-2 expression and increasing the BCL-2/Bax ratio in tumor cells [113]. On the other hand, the upregulation of PDGF and TGF-β are usually associated with increased expression of VEGF and bFGF in cancer cells [114]. MSCs also produce IL-6 that causes tumor cells to resistance to chemotherapy through upregulation of BCL-2 and activation of STAT-3 [113, 115]. STC-1 and NO (in low concentration) can inhibit apoptosis in tumor cells and promote tumor cell survival [113].

Anna and colleagues demonstrated that MSCs isolated from the umbilical cord (HUCMSCs) enhanced normal proliferation and migration of skin fibroblasts and promoted wound healing in the murine model through paracrine signaling [116]. Recently, Li et al. reported that in vivo transplantation of hAMSCs and hAMSC-CM considerably improved re-epithelialization and wound healing through enhancing proliferation and inhibiting apoptosis of heat-injured skin cells. The authors speculated that activation of PI3K/AKT and GSK3β/β-catenin signaling pathways might be responsible for the therapeutic impacts of hAMSCs/hAMSC-CM on the damaged skin cells [40]. Numerous studies have shown that hAMSCs contribute in activation of PI3K/AKT cell survival pathway by secreting different cytokines such as PAI-1, C-GSF, IL-6, IL-8, IL-11, OPN, HGF, TGFb1, RBP4, ANG-1, ANG-2, FAP, Galectin-1, DcR3, Follistatin, IGF-2, and MCP-1 [117]. Periostin, one of the matricellular proteins, is capable to activate the PI3K/AKT signaling pathway in tumor cells by interacting with integrin molecules [40]. This protein also can increase the migration and proliferation of epithelial cells and dermal fibroblasts via upregulating AKT/mTOR signaling pathway [118, 119]. Cetinkaya et al. observed the decreased expression of p57 cell cycle inhibitory protein and PARP-1 apoptosis marker as well as increased expression of Ki67 and PCNA following hAMSC administration [120].

Tissue inhibitor of metalloproteinases-1 (TIMP-1) is a protein released by hAMSCs, showing proliferative and anti-apoptotic features [121]. In a renal carcinoma model, it was shown that EVs released from hWJ-MSCs triggered the growth and invasiveness of renal carcinoma cells (RCC) in a xenograft model of BALB/c nu/nu mice [122]. Recently, it is reported that hUCMSC-EVs contain a large amount of miR-410 that strongly stimulate the growth of lung adenocarcinoma cancer cells in a xenograft tumor model [123]. According to all the previous studies, it seems that hUCMC-EVs are involved in growth and progression of tumor cells. Therefore, the use of them as a carrier for oncology therapy (engineered hUCMSC-EVs) may be a promising therapeutic strategy.

### Role of hAMSCs/hAFMSCs in promoting metastasis

**Fig. 3:**
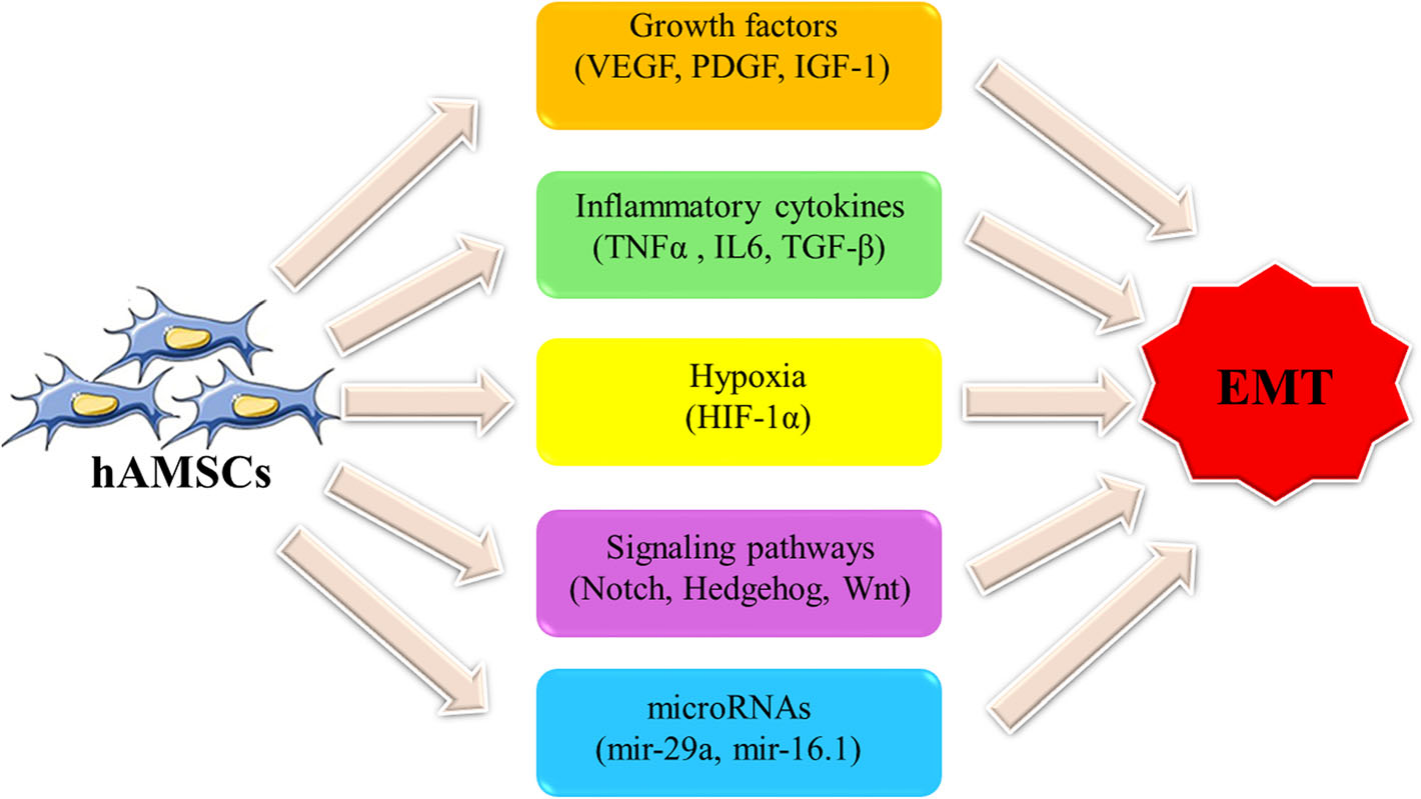
The role various effectors secreted by hAMSCs in induction of EMT. The mediators that promote EMT in epithelial cells include a wide list ranging from proteins to non-coding RNAs

Epithelial-mesenchymal transition (EMT) is a biological phenomenon in which epithelial cells acquire mesenchymal cell features. It plays a crucial role in cancer progression including invasiveness, metastasis, and the acquisition of resistance to apoptosis and chemotherapy [124–126]. There is an intertwined network of biological signaling pathways involved in the EMT process including Notch, Hedgehog, TGF-β/Smad, NF-κB, Wnt, and STAT3 pathways [127, 128]. Moreover, the EMT process can be facilitated by the presence of reactive oxygen species (ROS) and inflammatory mediators [129]. More recently, it has been indicated that cross-talk between MSCs and tumor cells increases the metastatic potential and EMT in these cells [130, 131]. Accordingly, hAMSCs by secreting a variety of paracrine factors such as FGF, EGF, VEGF, and, PDGF can significantly enhance or promote the EMT process (Fig. 3) [132]. In addition, studies have verified the presence of various types of MVs secreted by hAMSCs that take a role in this context [133]. It is found that these vesicles and their cargos play a key role in downregulation of MMP-1, MMP-13, TNF-α, IL-6, IL-1β genes, and the restoration of TGF-β expression [133, 134].

## Conclusion

**Fig. 4:**
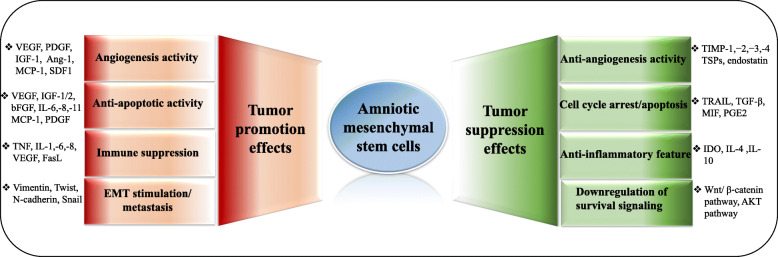
Schematic illustration of the dichotomy of hAMSCs in tumor progression. As shown in the figure, there is ample of evidence supporting both the contradictory effects of hAMSCs on cancer cells. These opposite effects are mediated by various cytokines and growth factors produced by these cells

Human amniotic mesenchymal stem cells act as a double-edged sword where they show either anti or protumorigenic effects (Fig. 4).

hAMSCs/hAFMSCs appear to be tumor promoter because these cells stimulate cancer cells’ growth, proliferation, invasion, metastasis, and tumor angiogenesis. On the other hand, there are many reports confirming the tumor suppressor effect of hAMSCs/hAFMSCs. Given that these cells exhibit unique features including stable characteristics, low immunogenicity, and nontumorigenicity, they are an attractive candidate to use in the clinic. However, conflicting results about the effect of these cells on tumors have cast doubt on their clinical applications.

Despite unpredictable behaviors of these cells in response to different cancer types, researchers can benefit from genetically modified hAMSCs/hAFSCs to bold anticancer side of these cells in cancer therapy. These genetically modified MSCs could be developed by two main strategies including overexpression of antitumorigenic mediators (interferons, interleukins, chemokines, and proapoptotic molecules) and encoding prodrugs or gene-directed enzymes in these cells. Moreover, hAMSC/hAFSC-derived exosomes can be used directly or as a carrier for delivering different anticancer drugs for treatment of different tumors.

## Data Availability

Not applicable.

## Abbreviations

CM: Conditioned medium
CTL: Cytotoxic T lymphocyte
CXCR4: Chemokine (C-X-C motif) Receptor type 4
IDO: Indoleamine 2,3 dioxygenase
HGF: Hepatocyte growth factor
HIFs: Hypoxia-inducible factors
hAMSCs: Human amniotic MSCs
hAFMSCs: Human amniotic fluid MSCs
hUCMSCs: Human umbilical cord MSCs
hUCMSC-EVs: hUCMSC-derived extracellular vesicles
PI3K: Phosphoinositide 3-kinase
TRAIL: TNF-related apoptosis-inducing ligand
MSCs: Mesenchymal stem cells
NO: Nitric oxide
SDF1: Stromal cell-derived factor-1
TGF-β: Transforming growth factor-beta
TNF-α: Tumor necrosis factor-alpha
